# Tolerability and adherence to adjuvant abemaciclib in hormone receptor-positive, HER2-negative, node-positive high-risk early breast cancer: a real-world study

**DOI:** 10.2340/1651-226X.2026.45961

**Published:** 2026-09-02

**Authors:** Carl Rosander, Karolina Larsson, Per Karlsson

**Affiliations:** Department of Oncology, Institute of Clinical Sciences, Sahlgrenska Academy, Sahlgrenska University Hospital, University of Gothenburg, Gothenburg, Region Vastra Gotaland, Sweden

**Keywords:** Breast cancer, HR-positive, abemaciclib, tolerability, real-world data, dose titration, healthcare utilization

## Abstract

**Background and purpose:**

Abemaciclib in addition to adjuvant endocrine therapy for hormone receptor positive (HR+) breast cancer improves disease free survival (IDFS) and overall survival (OS). However, treatment-related adverse events may lead to early discontinuation. Sahlgrenska University Hospital implemented a gradual increase in initial dosing, with the intention to improve tolerability and adherence.

**Patient/material and methods:**

This retrospective observational study described adherence, tolerability and healthcare utilization associated with a gradual dose increase of adjuvant abemaciclib. A total of 69 patients aged 31–81 years were treated at Sahlgrenska University Hospital between 1st of April 2023 and 1st of April 2025 with cut-off September 5th, 2025. Clinical encounters and treatment outcomes were extracted from medical records and analyzed using descriptive statistics.

**Results:**

Patients were older and had worse Eastern Cooperative Oncology Group status compared with the monarchE registrational trial. At 12 and 26 weeks, 34 patients (49%) and 25 patients (36%) remained on the full dose of abemaciclib, respectively. Overall, 65 patients (94.2%) and 58 patients (84%) remained on treatment at any dose at 12 and 26 weeks. Treatment discontinuation was mainly driven by low grade adverse events (grade 1–2). During the study period 1069 clinical encounters were recorded; 15 patients (22%) had at least one emergency care encounter and seven (10%) required hospital admission.

**Interpretation:**

The gradual dose increase strategy resulted in fewer patients maintaining the full dose at 12 weeks compared with monarchE and TRADE trials, while overall treatment persistence remained high. Treatment discontinuation was primarily driven by cumulative low-grade toxicity and healthcare utilization was considerable.

## Introduction

Abemaciclib in combination with endocrine therapy is an established adjuvant treatment for patients with HR+, Human epidermal growth factor negative (HER2-), node-positive, high-risk early breast cancer (eBC) [1, 2]. Abemaciclib is a cyclin-dependent kinase 4 and 6 (CDK4/6) inhibitor, which prevents cell-cycle progression from the G1-phase to the S-phase [3]. The pivotal monarchE trial demonstrated that the addition of abemaciclib, administered at a starting dose of 150 mg twice daily for 2 years, significantly improved disease-free survival (IDFS) and overall survival (OS) in patients with high risk breast cancer [4, 5]. Despite its proven efficacy, treatment-related adverse events may limit patients’ ability to complete the planned 2-year treatment course [1, 2, 6].

The phase II TRADE trial assessed a gradual dose increase strategy up to standard dose indicating an improvement of adherence at 12 weeks compared to the monarchE trial [1, 7]. However, it remains unclear how such dose increase strategy performs in routine clinical practice. Furthermore, real-world data on healthcare utilization associated with adjuvant abemaciclib treatment is limited.

This study aimed to describe the adherence and tolerability of the current administration plan with a gradual dose increase of abemaciclib treatment as an adjuvant treatment in patients with high-risk luminal breast cancer at Sahlgrenska University Hospital. This strategy was implemented based on the assumption that the treatment would be better tolerated, thereby improving adherence. Furthermore, the study aims to investigate healthcare utilization associated with abemaciclib and reasons for treatment discontinuation.

## Method

### Study design

This was a single-center, retrospective, observational study including patients from the Greater Gothenburg region who received adjuvant abemaciclib at Sahlgrenska University Hospital. Using the electronic booking system, 694 patients who had initiated any medical treatment for breast cancer at the Department of Oncology at Sahlgrenska University Hospital between April 1, 2023, and April 1, 2025, were identified. These patients were then manually reviewed to ensure that all patients with non-metastatic eBC who had initiated adjuvant abemaciclib were included in the analysis.

## Treatment protocol

The initial gradual dose increase protocol was implemented in April 2023 and consisted of abemaciclib 50 mg twice daily for 2 weeks, followed by 100 mg twice daily for another 2 weeks and finally to 150 mg twice daily if tolerated. From February 2024 and onwards, the starting dose was adjusted to 100 mg twice daily, as the initial experience indicated that 50 mg was associated with minimal toxicity and few adverse events. No major differences were observed between the patients who initiated treatment at 50 mg and those who initiated treatment at 100 mg twice daily with regard to baseline characteristics, adherence or toxicity. A detailed comparison between the two subgroups is presented in the Supplementary material.

Laboratory values and diarrhea were graded according to the European Medicine Agency guidelines [8].

### Data collection

Pathology reports provided data on ER, Progesterone receptor (PR), Ki-67, tumor grade, luminal A or B subtype, TNM classification and disease stage. Information on prior chemotherapy, endocrine therapy and radiotherapy was also collected. Eastern Cooperative Oncology Group (ECOG) performance status was obtained from the visit preceding abemaciclib initiation. Missing data were reported as ‘.’. Menopausal status was taken from the medical record and classified using a 50-year age threshold when it was unclear.

Data on clinical encounters and treatment outcomes were extracted from electronic medical records. The first recorded encounter corresponded to the consultation with an oncologist at which the treatment plan was established. All subsequent encounters with both oncologists and nurses were included, except for visits solely for prescription renewals or administration of adjuvant zoledronic acid. For each encounter, the mode of contact (in person, telephone, electronic) and the healthcare professional involved (nurse or oncologist) were recorded. Data collection ended at treatment discontinuation, disease recurrence or completion of the 2-year treatment period. For each encounter, information on continuation, current dose level, laboratory results, adverse events, and use of supportive medications was recorded. All emergency visits and hospital admissions reported to the oncology department were collected and reasons for dose reductions or treatment discontinuation were documented.

### Outcomes

The primary endpoint was the proportions of patients who reached and maintained the full dose of abemaciclib of 150 mg twice daily at 12 weeks. Secondary endpoints included the proportions of patients who reached and maintained the full dose at 26 weeks, as well as the proportions of patients who remained on treatment at any dose at 12 and 26 weeks. Additional secondary outcomes included reasons for treatment discontinuation and the healthcare utilization associated with abemaciclib, including the number of clinical encounters and communication modalities.

### Statistical analysis

For the descriptive analyses, STATA version 18 and R version 4.5.2 were used. Additional analyses such as logistic regression were performed but are presented in the Supplementary material to maintain focus in the main manuscript.

### Ethical considerations

Ethical approval was obtained from Swedish Ethical Review Authority (approval number 2024-08235-01). Personal data were stored on a secure research platform and anonymized prior to analysis.

## Results

### Baseline characteristics and study population

**Figure 1:**
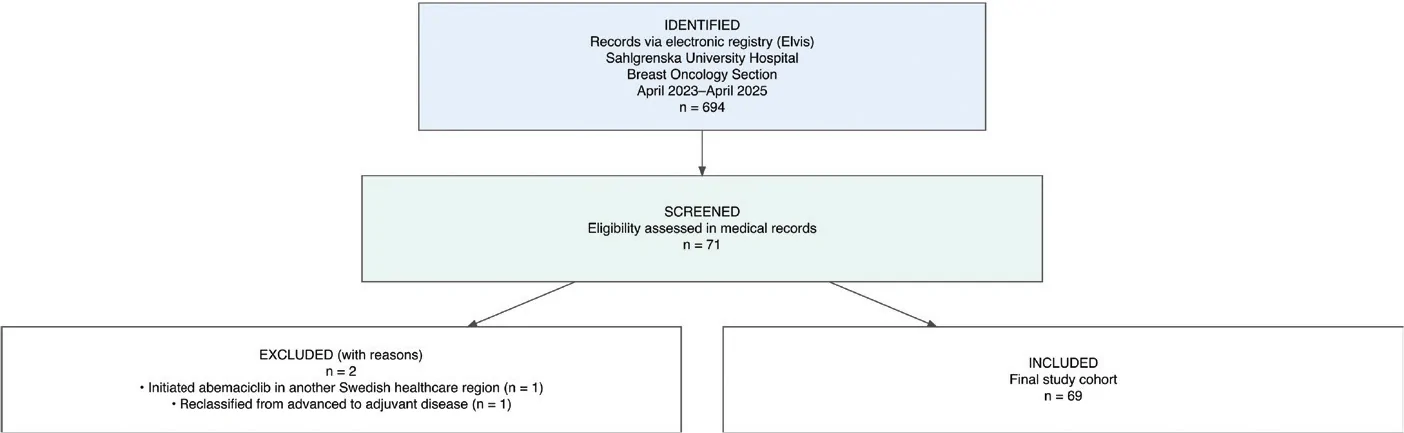
Flowchart illustrating the identification, screening and selection of the study cohort.

Baseline characteristics of the study population are presented in Table 1. A total of 69 patients were included in the study after exclusion of two patients. One had initiated abemaciclib treatment in another healthcare region in Sweden, and the other patient was reclassified from metastatic breast cancer to adjuvant disease (Figure 1). All patients were women, aged 31–81 years, with a median age of 57.5 years at treatment initiation. Most patients were postmenopausal (63.3%) and had an ECOG performance status 0 (71%). The median follow up time was 492 days (standard deviation [SD] = 191). Most tumors were classified as luminal B-like and almost all patients had received chemotherapy prior to initiation of abemaciclib. All patients received radiation therapy according to standard clinical practice.

**Table 1:** Baseline characteristics of patients treated with adjuvant abemaciclib at Sahlgrenska University Hospital.

Characteristic	*n* (%) or median (95% CI)
**Age, years**	57.5
≤ 65 years	44 (64)
> 65 years	25 (36)
**Sex**	
Women	69 (100)
**Menopausal status**	
Premenopausal	25 (36.2)
Postmenopausal	44 (63.8)
**WHO performance status**	
0	49 (71)
1	17 (24.6)
2	1 (1.5)
3	2 (2.9)
**Clinical stage (neoadjuvant subgroup)**	
2a	4 (23.5)
2b	5 (29.4)
3a	7 (41.2)
3b	1 (5.9)
**Pathological T**	
2a	7 (10.1)
2b	12 (17.4)
3a	40 (58)
3b	1 (1.5)
3c	9 (13)
**Luminal status**	
Luminal A – like	17 (24.6)
Luminal B – like	52 (75.4)
**Type**	
Ductal carcinoma	53 (76.8)
Lobular carcinoma	16 (23.2)
**NHG-grade**	
1	2 (2.9)
2	40 (58)
3	27 (39.1)
**KI67 index**	
Low (≤ 5%)	10 (14.5)
Intermediate (6–29%)	41 (59.4)
High (≥ 30%)	18 (26.1)
**Chemotherapy**	
Adjuvant chemotherapy	46 (66.7)
Neoadjuvant chemotherapy	17 (24.6)
No chemotherapy	6 (8.7)
**Radiation therapy**	
Yes	69 (100)
**Endocrine therapy**	
Aromatase inhibitor	49 (71)
Tamoxifen	20 (29)
GnRH-agonist	22

For patients treated with neoadjuvant chemotherapy, both clinical and pathological staging are reported in the table. A total of 22 patients received GnRH agonists in combination with endocrine therapy, among them 12 were treated with tamoxifen and 10 with aromatase inhibitors.

GnRH: gonadotropin-releasing hormone; NHG: Nottingham Histologic Grade.

### Adherence at 12 and 26 weeks

**Figure 2:**
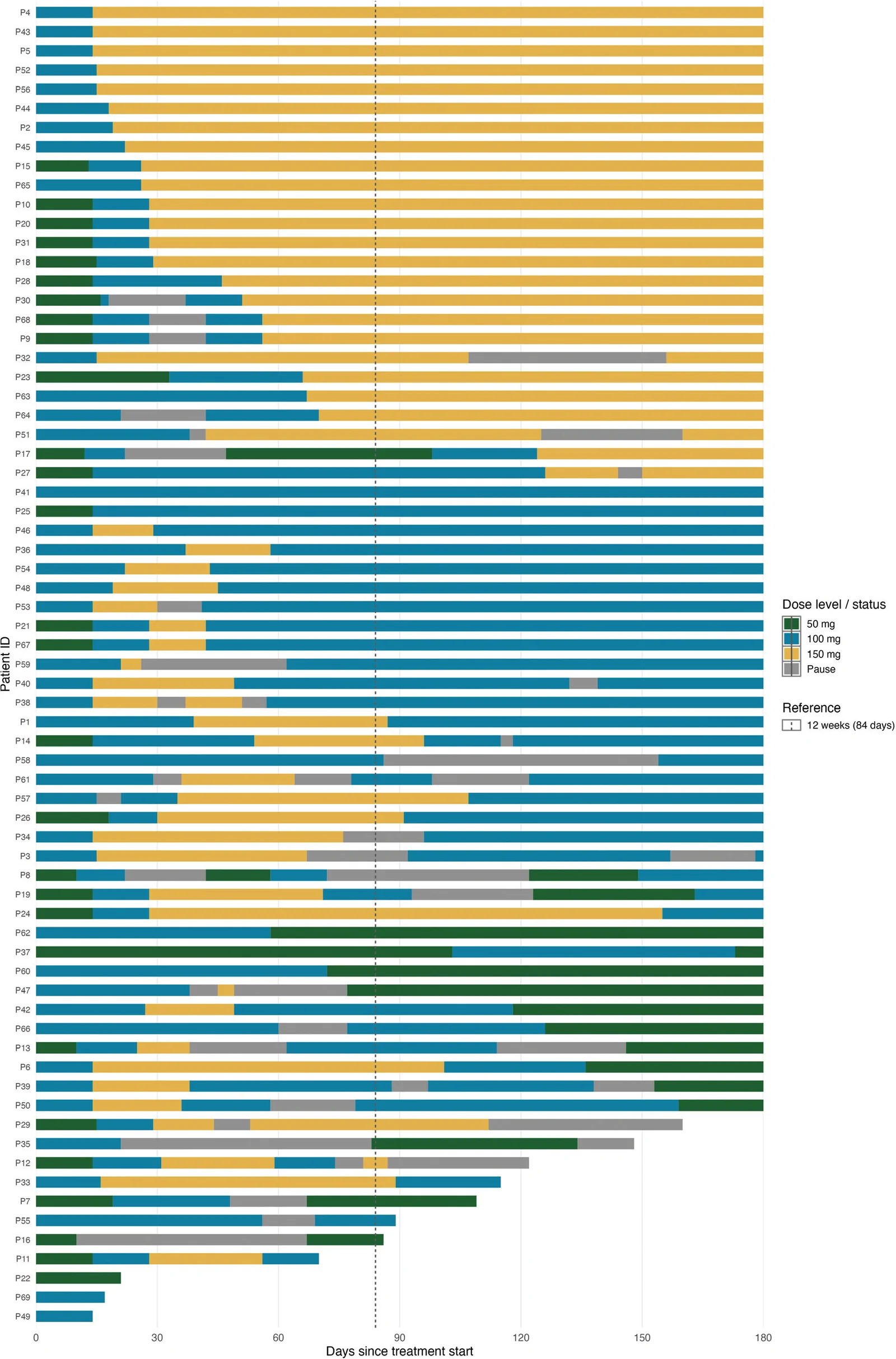
Swimmer´s plot illustrating individual dose levels of abemaciclib during the first 26 weeks of treatment. Green illustrates the dose 50 mg, blue 100 mg and yellow 150 mg. The dotted line shows the dose at 12 weeks.

The primary endpoint showed that 34 of 69 patients (49.3%) had reached and maintained the full abemaciclib dose of 150 mg twice daily at 12 weeks. In total, 65 patients (94.2%) remained on treatment at any dose. Three patients experienced a temporary treatment interruption at the 12-week assessment, two patients receiving 150 mg and one patient receiving 100 mg. At 26 weeks, 25 patients (36.2%) maintained the full abemaciclib dose of 150 mg twice daily and 58 patients (84.1%) remained on treatment at any dose (Table 2, Figure 2).

**Table 2:** Treatment status and healthcare utilization at 12 and 26 weeks among patients receiving adjuvant abemaciclib.

Outcome	12 weeks	26 weeks
Dose		
Discontinued treatment	4 (5.8)	11 (15.9)
50 mg	8 (11.6)	10 (14.5)
100 mg	23 (33.3)	23 (33.3)
150 mg	34 (49.3)	25 (36.2)
Encounters		
Number of encounters	564	797
Mean encounters per patient	8.2	11.5
Standard deviation	2	3.4

Data are presented as *n* (%) unless otherwise stated. Encounters refer to follow-up contacts between patients and healthcare professionals.

### Reasons for treatment discontinuation

By the data cut-off (September 5, 2025), 18 patients had discontinued treatment: 16 because of toxicity or adverse events and two following disease recurrence. The most common reason for discontinuation was general discomfort, followed by organ-related toxicities. Six patients reported more than one reason for discontinuation, most frequently the combination of diarrhea and general discomfort.

### Safety

**Figure 3:**
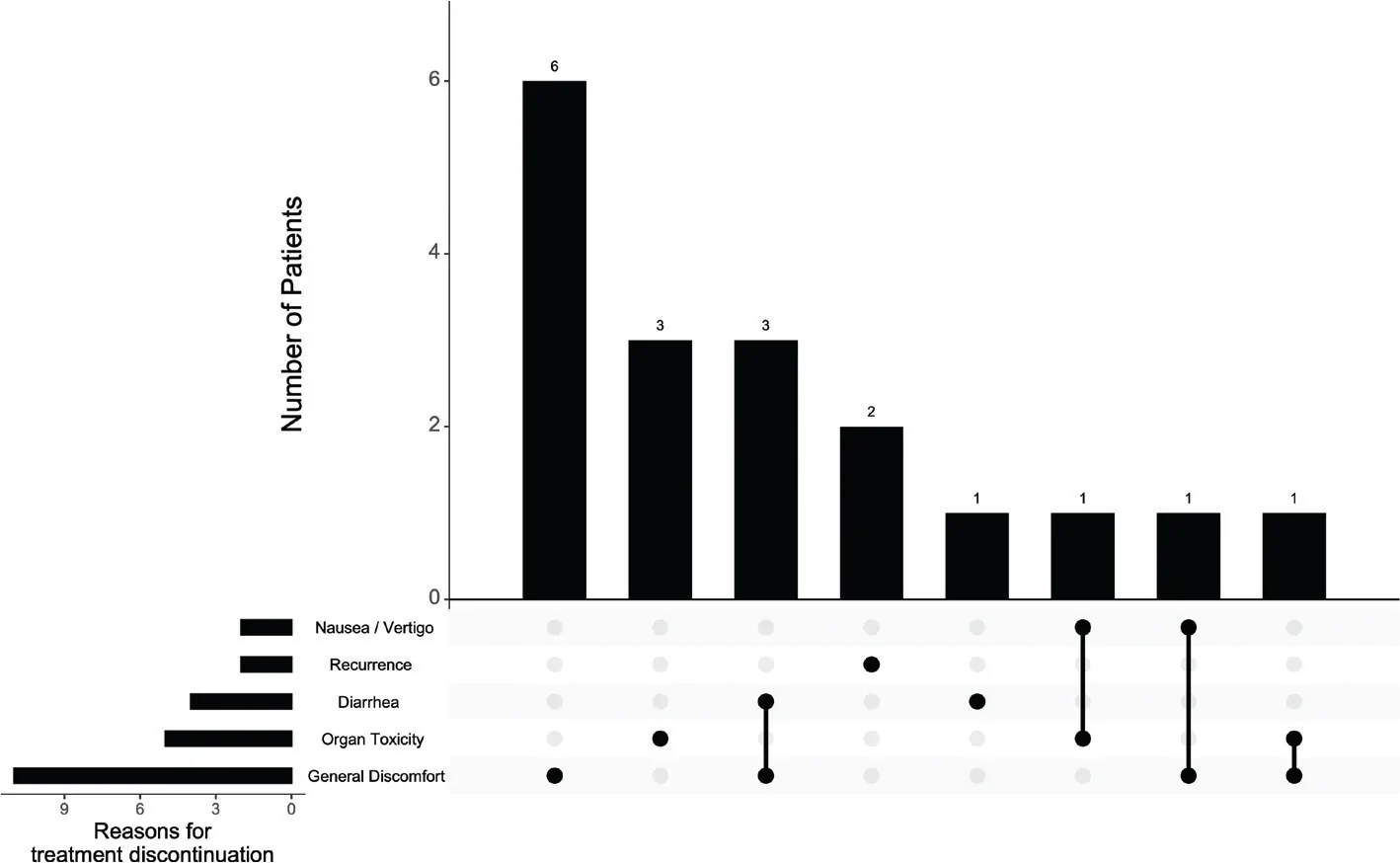
Upset plot. Illustrates the reasons for treatment discontinuation. Each bar represents the number of patients for each reason for treatment discontinuation. Connected points show combination of overlapping reasons.

**Figure 4:**
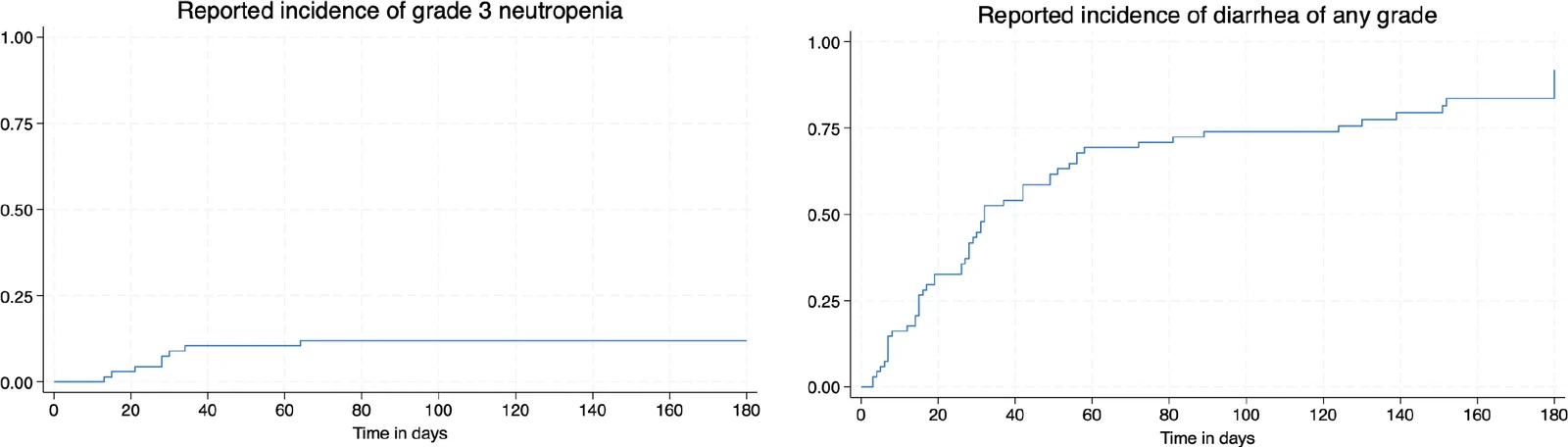
Kaplan–Meier failure curves. Reported incidence of diarrhea of any grade and grade 3 neutropenia in the first 26 weeks. The x-axis shows time on treatment (days), and the y-axis shows the cumulative incidence of the event.

Adverse events occurred early during treatment and were predominantly low grade (Figures 3 and 4). The most frequently reported adverse events of any grade were diarrhea (84.1%), fatigue (82.6%) and nausea (58%) (Table 3). Treatment interruptions and dose reductions occurred in 63.8% and 60.1% of patients, respectively.

**Table 3:** Safety outcomes and healthcare utilization at cut-off (September 5th, 2025) in patients treated with adjuvant abemaciclib.

Outcome	*n* (%) or summary measure
**Follow-up time in days**	
Mean per patient	492
Standard deviation	191
**Type of encounter**	
Nurse	819 (76.6)
Oncologist	183 (17.1)
Both	67 (6.3)
**Form of communication**	
Total	1069
Telephone	846 (79.1)
Electronic communication	52 (4.9)
Physical appointments	171 (16)
**Emergency care encounters**	
0	54 (78.3)
1	10 (14.5)
2	2 (2.9)
3	1 (1.5)
4	2 (2.9)
**Hospital admission**	
0	62 (89.9)
1	7 (10.1)
**Nights at the hospital**	
1	1 (14.3)
2	2 (28.6)
3	3 (42.9)
5	1 (14.3)
**Itching or eruption**	
No	57 (82.6)
Yes	12 (17.4)
**Venous thromboembolism**	
No	63 (91.3)
Yes	6 (8.7)
**Report of side effect at least once of any grade**	
Fatigue	57 (82.6)
Nausea	40 (58)
**Highest reported toxicity grade in the first 26 weeks**	
**Diarrhea (grade)**	
Grade 0	11 (16)
Grade 1	47 (68)
Grade 2	10 (14)
Grade 3	1 (1)
**Neutropenia (grade)**	
Grade 0	34 (49)
Grade 1	6 (9)
Grade 2	21 (30)
Grade 3	8 (12)
**Liver enzyme elevation (grade)**	
Grade 0	51 (74)
Grade 1	13 (19)
Grade 2	3 (4)
Grade 3	2 (3)
**Reported use of supportive medications at least once**	
metoclopramide	14 (20.3)
loperamide	45 (65.2)
**Dose at cut-off**	
0	16 (23.2)
50	17 (24.6)
100	16 (23.2)
150	18 (26.1)
Recurrence	2 (2.9)
**Number of treatment interruptions**	
0	25 (36.2)
1	19 (27.5)
2	15 (21.7)
3	6 (8.7)
4	2 (2.9)
5	1 (1.5)
8	1 (1.5)
**Length of treatment interruptions in days**	
Mean for patients that had a treatment interruption	40.98
Standard deviation	34.9
**Number of dose reductions**	
0	27 (39.1)
1	26 (37.7)
2	15 (21.7)
3	1 (1.5)

Venous thromboembolism (VTE) was observed in six patients, including two cases of pulmonary embolisms, both occurring in patients receiving aromatase inhibitors in combination with abemaciclib. Of the remaining four cases of VTE, two occurred in patients receiving aromatase inhibitors and two in patients receiving tamoxifen. Grade 3 neutropenia occurred in eight patients; all were younger than 65 years with ECOG performance status of 0.

### Healthcare utilization

Observed healthcare utilization exceeded that expected from the standard follow-up schedule. For patients who discontinued treatment early, the expected number of encounters was adjusted to their actual treatment duration. By week 26, a total of 797 clinical encounters were recorded, compared with 539 encounters expected according to routine follow-up, corresponding to a 48% higher number of contacts than anticipated.

By the cut-off (September 5th, 2025), a total of 1069 clinical encounters were recorded, of which 76.6% were conducted exclusively by nurses. Most of the contacts (79.1%) occurred via telephone. In total, 15 patients (21.8%) had at least one emergency contact, and seven patients (10.1%) required hospital admission for some reason. Two patients got admitted because of VTE, two for pneumonitis, two for neurological symptoms and lastly one for transient ischemic attack (TIA).

## Discussion and conclusion

### Comparison with other studies

In this observational study of patients with HR+, HER2-negative, eBC receiving adjuvant abemaciclib with a gradual dose increase, 49% of patients reached and maintained the full dose of abemaciclib at 12 weeks. This proportion was lower than reported in the TRADE study (70.8%) and in monarchE (60%) [7]. Direct comparisons between these studies should be interpreted with caution, as differences in baseline characteristics may have contributed to the observed differences in treatment outcomes. In our study, 70% of patients had a performance status of 0, compared with 91% in the monarchE, while performance status in TRADE was incompletely reported. In addition, our cohort included a lower proportion of patients younger than 65 years [1, 7]. Higher age and worse performance status have previously been associated with higher rates of treatment discontinuation and may therefore have influenced the observed outcomes [9].

Based on our clinical experience, most patients who initiated abemaciclib at 50 mg successfully escalated to 100 mg with few clinically relevant adverse events during the initial treatment phase. Consequently, the initial 50 mg step was considered unnecessary, and from February 2024, the starting dose was subsequently changed to 100 mg. This strategy remains the current clinical practice at Sahlgrenska University Hospital.

Since the introduction of adjuvant abemaciclib in HR+, HER2-, eBC another CDK4/6 inhibitor, ribociclib, has entered clinical practice following the positive results of the NATALEE-trial [10]. Whether a dose-escalation strategy could similarly improve tolerability and adherence remains to be determined.

The initial monitoring schedule at Sahlgrenska University Hospital was largely similar to that used in monarchE. However, in the monarchE trial follow-up visits were conducted in person, continued beyond the first 4 months, and followed a standard protocol [1]. The TRADE study does not present its monitoring schedule in detail, limiting direct comparisons. However, based on the available data, differences in follow-up intensity during the first 12 weeks alone are unlikely to explain the lower proportion of patients maintaining full dose in our cohort.

### Treatment discontinuation and safety

In both our cohort and in the monarchE trial, treatment discontinuation was frequently driven by the cumulative burden of persistent low-grade toxicities, such as fatigue, concentration difficulties and diarrhea, rather than isolated grade 3–4 adverse events. Consistent with monarchE, most grade 3–4 toxicities occurred early during treatment [2]. Treatment discontinuation may represent a more clinically relevant outcome than maintenance of full dose, particularly as previous analyses have demonstrated no difference in efficacy between patients requiring dose reductions and those remaining on full dose [2, 6]. However, these findings may not fully apply to a gradual dose increase strategy, as not all patients reach the full dose before experiencing toxicity. Another important aspect is maintaining patients on endocrine therapy. Recent reports have highlighted that discontinuation of CDK4/6 inhibitors is associated with a higher risk of discontinuing endocrine therapy as well and potentially compromises the overall benefit of adjuvant treatment [11, 12]. The observations further demonstrate the need for strategies to help patients remain on treatment such as a gradual dose increase.

In our cohort, six patients experienced VTE, which is a higher proportion of patients compared to the monarchE [2, 6]. Apart from VTE, the toxicity was broadly consistent with that reported in the monarchE. The incidence of diarrhea and neutropenia of any grade was similar between the studies. In contrast, fatigue and nausea were reported more frequently in our cohort. Fatigue was mentioned at least once in 82.6% of patients and nausea in 58%, compared with 40.6% and 29.5%, respectively, in the monarchE [6]. These findings should be interpreted with caution. One possible explanation could be an older cohort with a poorer performance status in our study, reflecting greater burden of comorbidities as expected when outcomes in patients treated in routine clinical practice are compared with those of a selected patient population enrolled in a clinical trial.

### Healthcare utilization

Published studies evaluating healthcare resource utilization associated with CDK4/6 inhibitors have predominantly focused on the metastatic setting. While some cost-effectiveness analyses exist, they do not capture the everyday clinical workload [13, 14]. To our knowledge, no real-world studies have quantified healthcare utilization related to adjuvant abemaciclib.

In our study, the introduction of adjuvant abemaciclib was associated with a significant amount of healthcare resources, primarily through nurse-led follow-up. A high number of patient encounters were recorded and approximately 22% of patients had at least one emergency contact, while 10% required hospital admission during treatment. These findings indicate that, despite abemaciclib being generally regarded as a tolerable therapy, the cumulative burden of low-grade toxicities, dose interruptions and dose adjustments translates into considerable use of healthcare resources in routine clinical practice.

### Strengths and limitations

A major strength of this study is the comprehensive capture of clinical encounters, decreasing the amount of missing data. However, the relatively small sample size and the absence of a control group limit the robustness of the conclusions. Comparisons with external studies should therefore be interpreted cautiously, as differences in patient characteristics and follow-up structures may introduce potential confounding. In addition, some degree of interpretation during data extraction was unavoidable. By including all patients treated with abemaciclib, this study provides complementary evidence that reflects routine clinical practice.

## Conclusions

In this observational study of patients with gradual dose increase of adjuvant abemaciclib, fewer patients reached and maintained the full dose compared to the TRADE trial and the monarchE regimen. Nevertheless, treatment persistence during the early treatment phase was comparable with TRADE despite worse ECOG status and older patients in this observational study. Treatment discontinuation was primarily driven by persistent low-grade toxicities, underscoring the clinical need for accessible support and proactive toxicity management. The study highlights the notable healthcare resources required for safe delivery of adjuvant abemaciclib.

## Supplementary Material



## Data Availability

The data of this study contain sensitive patient information and cannot be shared in its entirety because of ethical and legal retractions. De-identified data may be available from the corresponding author upon request with appropriate approval.
