# Supplementary material for: Tolerability and adherence to adjuvant abemaciclib in hormone receptor-positive, HER2-negative, node-positive high-risk early breast cancer: a real-world study
Source: Acta Oncol. 2026 Sep 2;65:45961. doi: 10.2340/1651-226X.2026.45961 (PMC13563436; doi:10.2340/1651-226X.2026.45961)
Supplement: Supplementary file 1 [file AO-65-45961-s1.pdf]

Supplementary material has been published as submitted. It has not been copyedited, or typeset by Acta Oncologica

Appendix: Tolerability and Adherence to Adjuvant Abemaciclib in Hormone Receptor-positive, HER2-Negative, Node-positive High-Risk Early Breast cancer: A Real-World Study

Supplementary exploratory analysis
In addition to the primary analyses presented in the main manuscript, exploratory analyses were performed, describing potential associations to achieved dose and occurrence of adverse events. To maintain focus, the analyses were not included in the main manuscript.

Supplementary research questions
1. Is there an association between age, menopausal status and WHO performance status and the achieved dose of abemaciclib?

2. Is there an association between age, menopausal status and WHO performance status affect incidence of side effects diarrhea and neutropenia?

Predictors of treatment continuation at 26 weeks
In this logistic regression analysis, WHO performance status was the only variable significantly associated with treatment continuation at 26 weeks (Table S1). Patients with a performance status of 0 were more likely to remain on treatment. Being premenopausal and younger than 65 years showed better outcomes, but these factors were not statistically significant. The regression model explained approximately 9% of variance in treatment continuation, and the overall model was not statistically significant. The two patients with recurrence were still receiving abemaciclib at the time of the analysis.

Table S1. Logistic regression - on treatment after 26 weeks

**Variable**	**OR**	**95% CI**	**P-value**
**Menopausal status**	1.67	0.16–3.89	0.535
**Age**	0.54	0.10–2.98	0.481
**Performance status**	0.22	0.05–0.89	0.033

Table note. OR= odds ratio. CI = confidence interval.

Predictors of adverse events at 26 weeks
Grade ≥ 2 diarrhea
In the logistic regression analysis, none of the evaluated variables, menopausal status, age or WHO performance status were significantly associated with the occurrence of grade 2 and 3 diarrhea at 26 weeks (Table S2).

Grade 3 neutropenia
For grade 3 neutropenia, only menopausal status could be included in the logistic regression model. The variable was not significantly associated with the occurrence of neutropenia (Table S3). As all patients who developed grade 3 neutropenia had a performance status of 0 and were younger than 65 years, logistic regression could not be performed for these variables.

Table S2 - logistic regression - grade 2 diarrhea or more

Variable	OR	95% CI	P-value
**Menopausal status**	1.5	0.24-9.44	0.665
**Age**	0.58	0.13–2.62	0.478
**Performance status**	0.18	0.004-8.29	0.379

Table note. OR= odds ratio. CI = confidence interval. Adverse events were defined as grade ≥ 2 diarrhea, corresponding to events leading to dose modification or treatment interruption.

Table S3 - logistic regression - grade 3 neutropenia

Variable	OR	95% CI	P-value
**Menopausal status**	2.57	0.44-5.2	0.297
**Age**	-	-	-
**Performance status**	-	-	-

Table note. Only menopausal status was included in the logistic regression. OR= odds ratio. CI = confidence interval. Adverse events were defined as grade ≥ 3 neutropenia, corresponding to events leading to dose modification or treatment interruption.

Comparison between patients who initiated treatment at 50 mg and 100 mg

Table S4 presents the baseline characteristics of patients who initiated adjuvant abemaciclib at 50 mg or 100 mg. Treatment status at twelve and 26 weeks is presented in Table S5 and S6, respectively. Table S7 summarizes follow-up time and healthcare utilization according to initial starting dose. Due to the limited sample size of the two subgroups, comparisons should be interpreted with caution. Overall, no major differences were observed between the groups regarding baseline characteristics, adherence, healthcare utilization or safety.

Table S4 - Baseline characteristics according to initial abemaciclib starting dose.

Characteristic	*50mg; n(%) or median (95% CI)*	*100mg; n(%) or median (95% CI)*
**Age, years**		
≤ 65 years	17 (60.7)	27 (65.9)
>65 years	11 (39.3)	14 (34.1)
**Sex**		
Women	28 (100)	41 (100)
**Menopausal status**		
Premenopausal	6 (21.4)	12 (29.3)
Postmenopausal	22 (78.6)	29 (70.7)
**WHO performance status**		
0	20 (71.4)	29 (70.7)
1	8 (28.6)	9 (22)
2	0	1 (2.4)
3	0	2 (4.9)
**Clinical stage (neoadjuvant subgroup)**		
2a	1 (14.3)	3 (30)
2b	3 (42.9)	2 (20)
3a	2 (28.6)	5 (50)
3b	1 (14.3)	0
**Pathological T**		
2a	3 (10.7)	4 (9.8)
2b	9 (32.1)	3 (7.3)
3a	12 (42.9)	28 (68.3)

3b	1 (3.6)	0
3c	3 (10.7)	6 (14.6)
**Luminal status**		
Luminal A - like	5 (17.9)	12 (29.3)
Luminal B - like	23 (82.1)	29 (70.7)
**Type**		
Ductal carcinoma	20 (71.4)	33 (80.5)
Lobular carcinoma	8 (28.6)	8 (19.5)
**NHG-grade**		
1	0	2 (4.9)
2	18 (64.3)	22 (53.7)
3	10 (35.7)	17 (41.5)
**KI67 index**		
Low (≤ 5%)	3 (10.7)	7 (17.1)
Intermediate (6-29%)	17 (60.7)	24 (58.5)
High (≥30%)	8 (28.6)	10 (24.4)
**Chemotherapy**		
Adjuvant chemotherapy	19 (67.9)	27 (65.9)
Neoadjuvant chemotherapy	7 (25)	10 (24.4)
No chemotherapy	2 (7.1)	4 (9.8)
**Radiation therapy**		
Yes	28 (100)	41 (100)
**Endocrine therapy**		
Aromatase inhibitor	19 (67.9)	30 (73.2)
Tamoxifen	9 (32.1)	11 (26.8)
GnRH-agonist	9 (32.1)	13 (31.7)

Table note. For patients treated with neoadjuvant chemotherapy, both clinical and pathological staging are reported in the table. GnRH =gonadotropin-releasing hormone), NHG =Nottingham Histologic Grade)

Table S5 - Treatment status and healthcare utilization at twelve weeks according to initial abemaciclib starting dose.

**Outcome,**	**50 mg start dose**	**100 mg start dose**
**Dose**		
Discontinued treatment	2 (7.1)	2 (4.9)
50 mg	4 (14.3)	4 (9.8)
100 mg	7 (25)	16 (39)
150 mg	15 (53.6)	19 (46.3)
**Encounters**		
Number of encounters	240	324
Mean encounters per patient	8.6	7.9
Standard deviation	2.3	2.1

Table note. Data is presented as n (%) unless otherwise stated. Encounters refer to follow-up contacts between patients and healthcare professionals.

Table S6 - Treatment status and healthcare utilization at 26 weeks according to initial abemaciclib starting dose.

**Outcome,**	**50 mg start dose**	**100 mg start dose**
**Dose**		
Discontinued treatment	6 (21.4)	5 (12.2)

50 mg	2 (7.1)	8 (19.5)
100 mg	8 (28.6)	15 (36.6)
150 mg	12 (42.9)	13 (31.7)
**Encounters**		
Number of encounters	336	461
Mean encounters per patient	12	11.2
Standard deviation	3	3.6

Table note. Data is presented as n (%) unless otherwise stated. Encounters refer to follow-up contacts between patients and healthcare professionals.

Table S7 - Safety outcomes and healthcare utilization at cut-off (September 5th, 2025) according to initial abemaciclib starting dose.

Outcome	**50 mg start dose**	**100 mg start dose**
**Follow-up time in days**		
Mean per patient	678	366
Standard deviation	98	122
**Type of encounter**		
Nurse	329 (73.8)	490 (78.7)
Oncologist	90 (20.2)	93 (14.9)
Both	27 (6.1)	40 (6..4)
**Form of communication**		
Total	446	623
Telephone	347 (77.8)	499 (80.1)
Electronic communication	20 (4.5)	32 (5.1)
Physical appointments	79 (17.7)	92 (14.8)
**Emergency care encounters**		
0	18 (64.3)	36 (87.8)
1	9 (32.1)	1 (2.4)
2	0	2 (4.9)
3	0	1 (2.4)
4	1 (3.6)	1 (2.4)
**Hospital admission**		
0	23 (82.1)	39 (95.1)
1	5 (17.9)	2 (4.9)
**Nights at the hospital**		
1	1 (20)	0
2	1 (20)	1 (50)
3	3 (60)	0
5	0	1 (50)
**Itching or eruption**		
No	22 (78.6)	35 (85.4)
Yes	6 (21.4)	6 (14.6)
**Venous thromboembolism**		

No	25 (89.3)	37 (90.2)
Yes	3 (10.7)	4 (9.8)
**Report of side effect at least once of any grade**		
Fatigue	20 (71.4)	37 (90.2)
Nausea	20 (71.4)	20 (48.8)
**Highest reported toxicity grade in the first 26 weeks**		
Diarrhea (grade)		
Grade 0	3 (10.7)	8 (19.5)
Grade 1	21 (75)	26 (63.4)
Grade 2	4 (14.3)	6 (14.6)
Grade 3	0	1 (2.4)
Neutropenia (grade)		
Grade 0	14 (50)	20 (48.8)
Grade 1	4 (14.3)	2 (4.9)
Grade 2	7 (25)	14 (34.1)
Grade 3	3 (10.7)	5 (12.2)
Liver enzyme elevation (grade)		
Grade 0	19 (67.9)	32 (78)
Grade 1	5 (17.9)	8 (19.5)
Grade 2	2 (7.1)	1 (2.4)
Grade 3	2 (7.1)	0
**Reported use of supportive medications at least once**		
metoclopramide	4 (14.3)	10 (24.4)
loperamide	17 (60.7)	28 (68.3)
**Dose at cut-off**		
0	9 (32.1)	7 (17.1)
50	5 (17.9)	14 (34.1)
100	5 (17.9)	9 (22)
150	8 (28.6)	10 (24.4)
Recurrence	1 (3.6)	1 (2.4)
**Number of treatment interruptions**		
0	7 (25)	18 (43.9)
1	13 (46.4)	6 (14.6)
2	5 (17.9)	10 (24.4)
3	2 (7.1)	4 (9.8)
4	1 (3.6)	1 (2.4)
5	0	1 (2.4)
8	0	1 (2.4)
**Length of treatment interruptions in days**		
Mean for patients that had a treatment interruption	29	51.9
Standard deviation	22	41
**Number of dose reductions**		

0	12 (42.9)	15 (36.6)
1	10 (35.7)	16 (39)
2	5 (17.9)	10 (24.4)
3	1 (3.6)	0

Figure S1 shows the highest reported toxicity grade during the first 26 weeks of treatment. Most patients experienced only grade low grade toxicity, which did not result in treatment interruption or dose reduction. Eleven patients (15%) developed grade ≥2 diarrhea, eight (12%) experienced grade 3 neutropenia, and five (7%) had ≥2 grade liver enzyme elevation leading to treatment interruption.

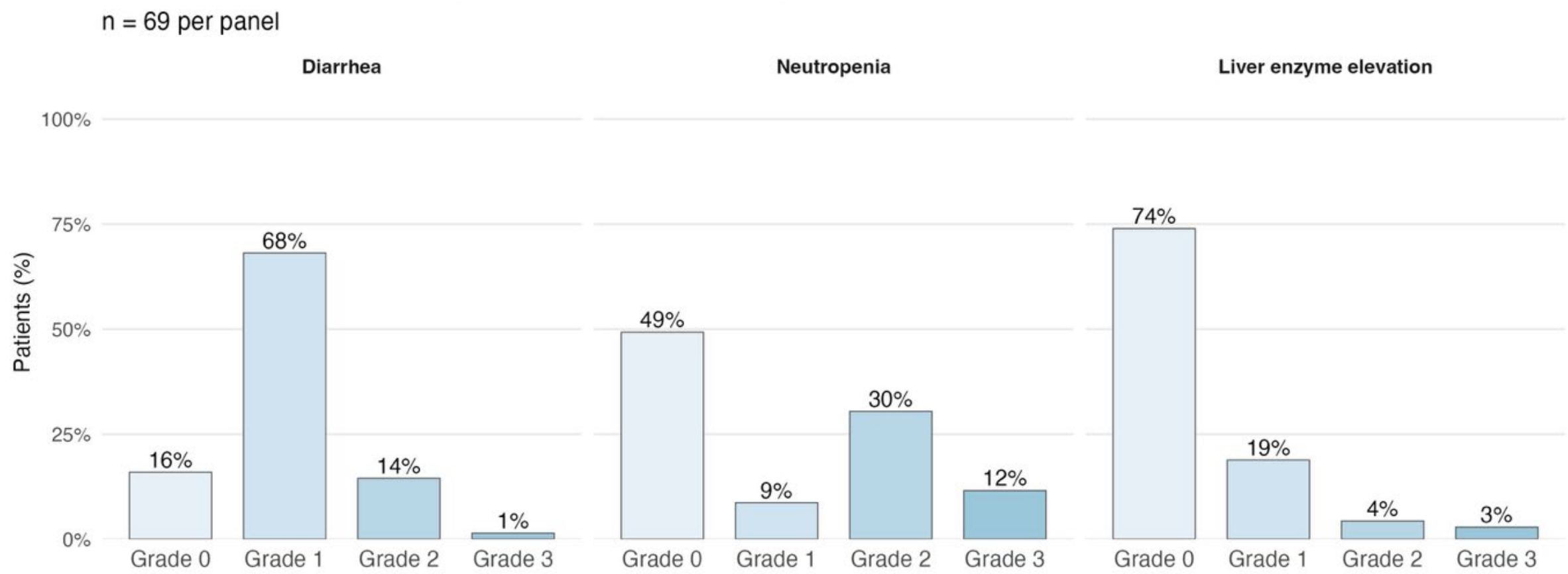

Figure S1: Highest reported toxicity grade in the first 26 weeks.

In Figure S2, the distribution of clinical encounters the first 26 weeks can be illustrated. Most patients have around ten encounters, but a distinct variation can be seen. It is important to keep in mind that the number of encounters is not adjusted to the expected number of clinical encounters. Patients with the fewer number of encounters and have discontinued treatment early and have therefore naturally fewer encounters.

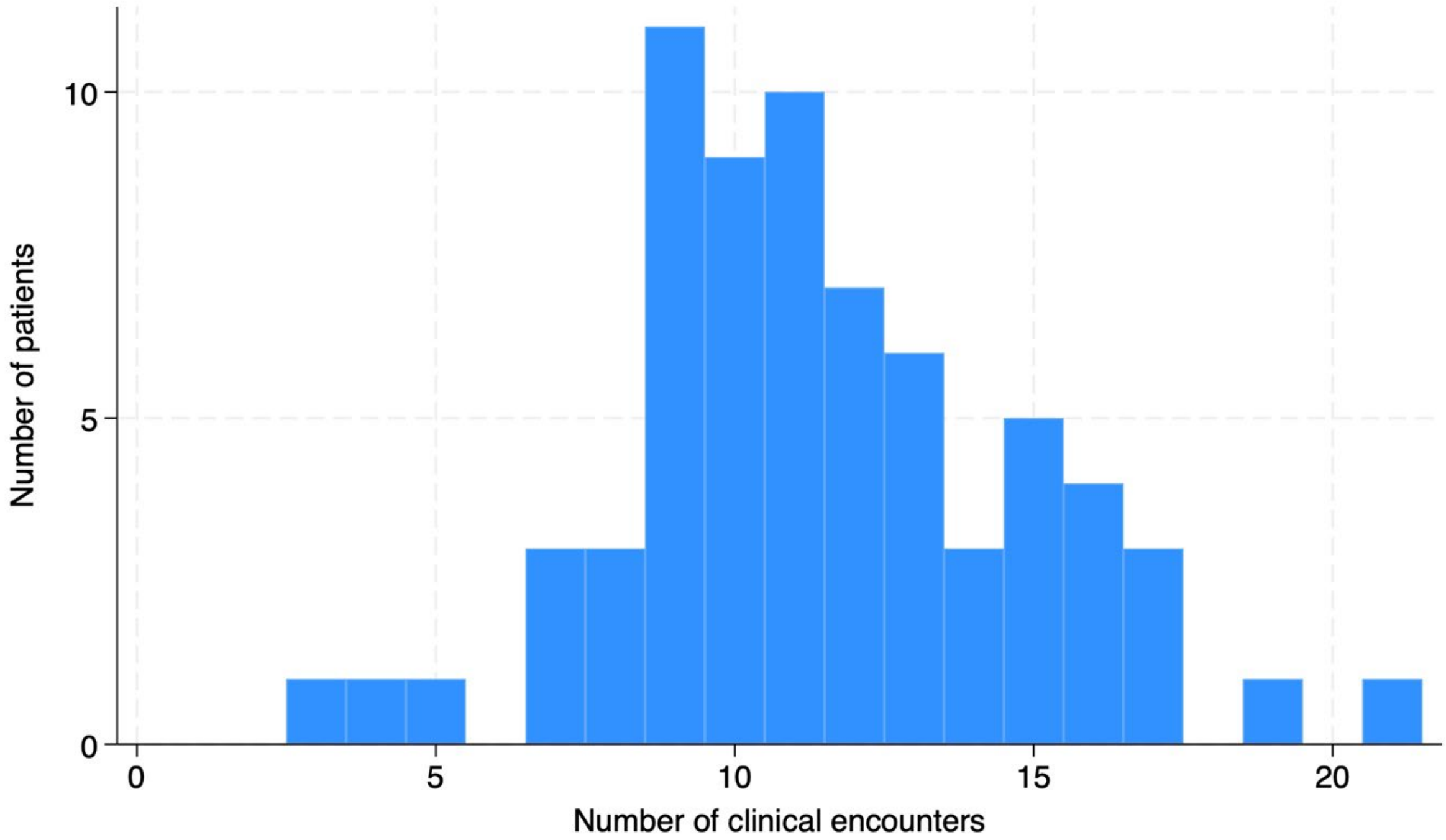

Figure S2: Distribution of the number of clinical encounters during the first 26 weeks of treatment.
